# A framework for prospective, adaptive meta-analysis (FAME) of aggregate data from randomised trials

**DOI:** 10.1371/journal.pmed.1003629

**Published:** 2021-05-06

**Authors:** Jayne F. Tierney, David J. Fisher, Claire L. Vale, Sarah Burdett, Larysa H. Rydzewska, Ewelina Rogozińska, Peter J. Godolphin, Ian R. White, Mahesh K. B. Parmar

**Affiliations:** MRC Clinical Trials Unit at UCL, Institute of Clinical Trials & Methodology, University College London, United Kingdom

## Abstract

**Background:**

The vast majority of systematic reviews are planned retrospectively, once most eligible trials have completed and reported, and are based on aggregate data that can be extracted from publications. Prior knowledge of trial results can introduce bias into both review and meta-analysis methods, and the omission of unpublished data can lead to reporting biases. We present a collaborative framework for prospective, adaptive meta-analysis (FAME) of aggregate data to provide results that are less prone to bias. Also, with FAME, we monitor how evidence from trials is accumulating, to anticipate the earliest opportunity for a potentially definitive meta-analysis.

**Methodology:**

We developed and piloted FAME alongside 4 systematic reviews in prostate cancer, which allowed us to refine the key principles. These are to: (1) start the systematic review process early, while trials are ongoing or yet to report; (2) liaise with trial investigators to develop a detailed picture of all eligible trials; (3) prospectively assess the earliest possible timing for reliable meta-analysis based on the accumulating aggregate data; (4) develop and register (or publish) the systematic review protocol before trials produce results and seek appropriate aggregate data; (5) interpret meta-analysis results taking account of both available and unavailable data; and (6) assess the value of updating the systematic review and meta-analysis. These principles are illustrated via a hypothetical review and their application to 3 published systematic reviews.

**Conclusions:**

FAME can reduce the potential for bias, and produce more timely, thorough and reliable systematic reviews of aggregate data.

## Abstract 2

Jayne Tierney and coauthors discuss FAME, an approach for adaptive meta-analysis of data from randomised trials.

## Background

The vast majority of systematic reviews are planned retrospectively, once most eligible trials have completed and reported, and are based on aggregate data extracted from publications. However, prior knowledge of trial results can introduce bias into both review and meta-analysis methods (Table 1), and the omission of unpublished data can introduce reporting biases [1]. Often unpublished and ongoing trials are overlooked [2,3], meaning results may not be interpreted in the context of all the potential evidence, and updating is considered separately [4]. A central tenet of randomised controlled trials is that they are designed prospectively, with methods specified prior to data analysis, in order to preserve objectivity and avoid bias, yet paradoxically, retrospective systematic reviews are often considered a higher level of evidence. Prospective meta-analysis (PMA) has been proposed as a “next generation” solution to the limitations [5].

**Table 1:** Aspects of conventional, retrospective systematic reviews and meta-analyses that can be biased by prior knowledge of trial results.

Aspect of conventional retrospective review	Potential Bias
Timing of systematic review or meta-analysis	Systematic differences between trial results available at the time of the review or meta-analysis, and the remaining eligible trials. This can occur, for example, if the review or meta-analysis coincides with the publication of striking trial results, and these are published first.
Choice of objective and trial eligibility criteria	Systematic differences between results of trials that are, and are not, selected for inclusion. This might arise if the objective and eligibility criteria selected are narrow, thereby excluding trials with particular results, for example, those that that don’t fit with prior beliefs.
Choice of participant eligibility criteria	Systematic differences between results for participants that are, and are not, selected for inclusion. This can be an issue, for example, if a treatment is (or appears to be) beneficial only in certain participant subgroups.
Choice of main outcome(s)	Systematic differences between results for outcomes that are, and are not, selected for inclusion. This can lead to bias, for example, if treatment benefits or harms are apparent for some outcomes and not others.
Assessment of risk of bias	Systematic differences in risk of bias assessment according to results. This can occur, for example, if trials with unexpected or discordant results are regarded as being at higher risk of bias.
Methods of analyses including: • Choice of model • Choice of subgroup analyses by trial or participant characteristics • Choice of sensitivity analysis by trial characteristics, risk of bias or results	Systematic differences between meta-analysis results according to methods of analysis. This could arise if, for example: • a random effects model is selected, giving larger weight to small trials with more pronounced effects; • the selection of participant subgroup variables or subgroup analyses is driven by subgroup interactions already observed in one or more trials; • a sensitivity analysis excludes trials with extreme results, but without other justification.

In PMA, all methods are planned before results of included trials are known, thereby limiting bias [6]. If a PMA is designed when eligible trials are ongoing, or being planned, investigators can work together to harmonise their trial designs, data collection, and other processes [6]. Even if a PMA is initiated when trials are near completion, bias can still be reduced, e.g., by standardising data definitions and analyses. To date, most PMAs have been based on individual participant data (IPD), which brings additional advantages, such as the ability to include more outcomes, standardise their definitions, and carry out more in-depth analyses, including investigation of subgroup effects [7,8]. However, the time lag between trial completion and availability of IPD precludes rapid evidence synthesis. Therefore, we have developed a Framework for Adaptive Meta-analysis (FAME) [9,10] that is prospective and collaborative in nature but uses aggregate data to provide results that are timelier and less prone to bias. Unlike “living” systematic reviews [11] that incorporate new trial evidence as it emerges, with FAME, we monitor how evidence from trials is accumulating, to anticipate the earliest opportunity for a potentially definitive meta-analysis. This paper outlines FAME and illustrates its application to systematic reviews in prostate cancer.

## Methodology

### Piloting FAME

In 2015, recognising that a number of trials investigating the effects of docetaxel and zoledronic acid for hormone-sensitive prostate cancer were due to produce results, we wanted to find a way to synthesise these in a timely and unbiased way, to quickly inform clinical practice and an ongoing, adaptive trial [12]. By engaging with investigators, we learned more about the design, conduct, analysis, and dissemination plans of the eligible trials. This allowed us to develop systematic review methods prior to most trial results being known (PROSPERO protocol CRD42015020059), and gauge how soon reliable meta-analyses might be possible. For example, we showed definitively that adding docetaxel to standard therapy improves the survival of men with advanced prostate cancer, whereas adding zoledronic acid does not, ahead of all trial results being available [13]. An unanticipated benefit was gaining access to trial results prepublication, speeding up the review process further. This pilot prompted us to be entirely prospective in the planning of subsequent reviews, and to routinely seek extra results from investigators to improve the quality and consistency of the analyses.

**Fig 1:**
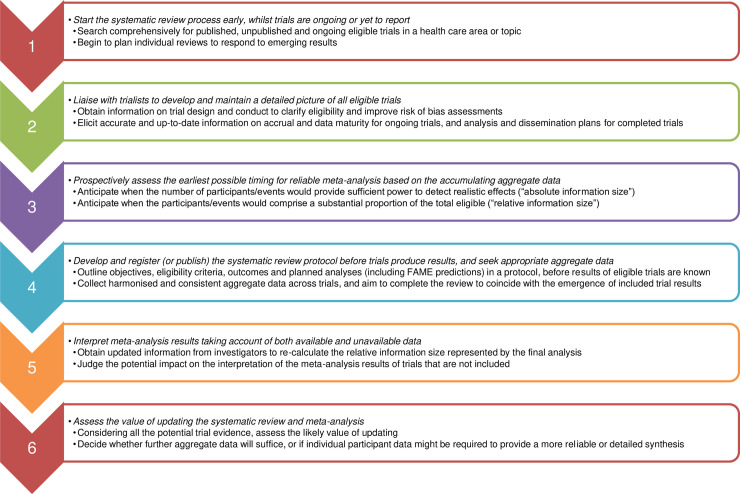
Framework for prospective, adaptive meta-analysis (FAME): Summary of key principles.

**Fig 2:**
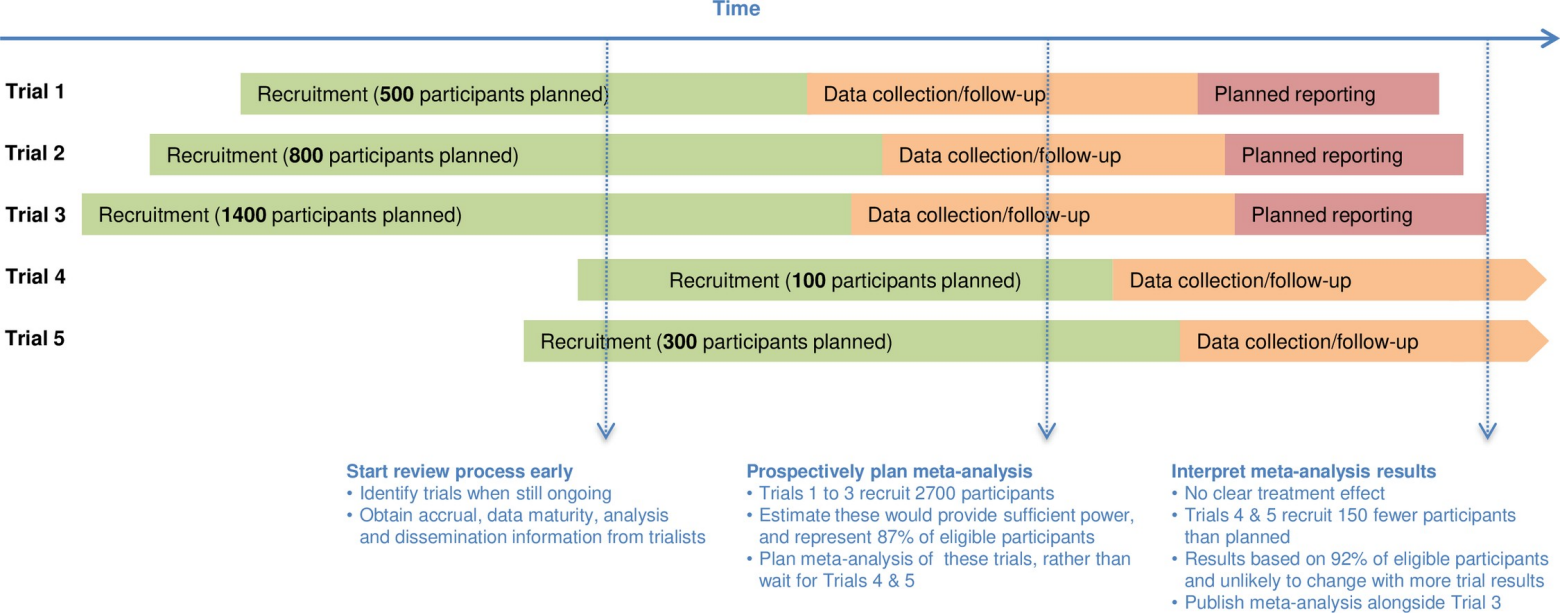
Visualising the application of FAME to a hypothetical systematic review of 5 randomised trials.

The refined principles of FAME are detailed below, summarised in Fig 1, and illustrated via a hypothetical review of 5 randomised trials (Fig 2).

### FAME key principles

1. *Start the systematic review process early*, *while trials are ongoing or yet to report*
  As for any PMA, the review process should be initiated when eligible trials are ongoing, or before their final analyses, to avoid the methods being biased by prior knowledge of trial results. Acquaintance with the healthcare area is advantageous for identifying unpublished and ongoing trials, but must be backed up by a comprehensive search [14]; with registers, regulatory agencies, and investigator networks being particularly crucial sources of trials. As depicted in Fig 2, at initiation of the hypothetical review, all 5 trials are ongoing.
2. *Liaise with trial investigators to develop a detailed picture of all eligible trials*
  Engagement with trial investigators is critical to obtaining the information needed for review planning, prioritisation and conduct. They can clarify aspects of trial design and conduct, which improves eligibility screening and the accuracy of risk of bias assessments [15]. Also, importantly, they can provide accurate and up-to-date information on accrual, data maturity, analysis, and dissemination plans without compromising the individual trials. Collaborating investigators become co-contributors to the review and coauthors on the final publication. For the hypothetical review, contact with investigators helps clarify that each trial is eligible and at low risk of bias.
3. *Prospectively assess the earliest possible timing for reliable meta-analysis based on the accumulating aggregate data*
  Evidence suggests that more reliable results for overall treatment effects are obtained when the total number of participants or events (“absolute information size”) and the proportion of eligible participants or events (“relative information size”) included in aggregate data meta-analyses are large [16]. Knowledge garnered from trial investigators can be used to estimate the absolute and relative information size of the accumulating aggregate data, and therefore anticipate when there will be enough for a reliable meta-analysis. Such reliable evidence synthesis may be achieved months or years before all eligible trial results are available.
  Firstly, there is a need to determine if, and when, the accumulating absolute information would likely provide sufficient power to detect realistic and clinically meaningful effects of the intervention under investigation. Provided care is taken to minimise potential heterogeneity when specifying the objective and eligibility criteria, the meta-analysis can be regarded like a single prospective trial, in which the accumulating information is monitored to determine the optimum timing of the final analysis, blinded to the results. As such, standard sample size methods and typical control-group event rates for the particular population are used, and these target magnitudes of effect not larger than those being targeted in the included trials. From our experience in cancer, relative risk reductions of around 20% to 25% (or absolute differences of 5% to 10%) are both realistic and worthwhile, but may be adapted for other healthcare areas. For binary and time-to-event and outcomes, the absolute information size will relate to the number of participants and events and, for continuous outcomes, to the number of participants. Additionally, for time-to-event outcomes, the follow-up will need to be sufficient for the population being studied.
  Secondly, there is a need to assess when the anticipated number of participants or events would comprise a large proportion of those potentially available from all eligible trials (whether completed or not). This is to ensure that conclusions are unlikely to be overturned later. For active trials, the potential number of participants may need to be estimated from current or planned accrual figures.
  Clearly, striking a balance between maximising the absolute and relative information size and producing a sufficiently timely review is an important consideration. For example, in the hypothetical review, the 3 largest trials (which are due to complete first) will likely provide sufficient power to detect an effect on the main outcome and constitute a substantial proportion of all potentially eligible participants (Fig 2). Thus, a meta-analysis of these 3 trials is planned to provide both an early and reliable synthesis.
4. *Develop and register (or publish) the systematic review protocol before trials produce results and seek appropriate aggregate data*
  To avoid bias, the objectives, eligibility criteria, outcomes, and planned analyses must be outlined in a publicly available protocol, before results of all (or most) eligible trials are known [6]. The FAME estimates of absolute information size, power and relative information size, and the associated decision on meta-analysis timing should be included. Rather than be bound by the planned individual trial analyses, there is the opportunity to agree with investigators, for example, new outcome and subgroup definitions and additional analyses, then collect aggregate data accordingly. This can improve the quality, reliability, and interpretability of meta-analysis results.
  If possible, review completion should be timed to coincide with the emergence of included trial results to provide the greatest potential for it to impact expeditiously on clinical practice and on related ongoing or planned trials. In the hypothetical review, the manuscripts for the meta-analysis and the largest trial are prepared in tandem, with a view to co-publication (Fig 2).
5. *Interpret meta-analysis results taking account of both available and unavailable data*
  Added to standard considerations such as the direction and precision of the overall effect, and any unanticipated heterogeneity, it is important to assess the potential impact of trials that were not included on the interpretation of the meta-analysis results. This relies on obtaining updated information on all eligible trials from investigators, reestimating the absolute information size, and from that, the relative information size represented by the data. The hypothetical review is based on a larger proportion of the evidence than originally anticipated, because the later trials did not recruit to target (Fig 2), and the results show no clear overall effect of treatment.
6. *Assess the value of updating the systematic review and meta-analysis*
  Considering all the potential trial evidence, whether included or not, also makes it possible to ascertain whether there is likely to be value in updating the meta-analysis with further aggregate data, or if IPD might be required for a more reliable or detailed synthesis [16]. This will depend, for example, on the direction and precision of the existing meta-analysis result, which trial results have yet to emerge, how quickly an answer is needed and the resources available. With little trial evidence still to emerge in the hypothetical review, updating the meta-analysis is considered to be of limited value.

## Implementation of FAME

We illustrate the application of FAME to 3 published reviews in prostate cancer:

### 1. Effects of adding abiraterone to standard care in metastatic prostate cancer

In 2016, we identified 3 trials evaluating the addition of abiraterone to standard hormone therapy for metastatic prostate cancer, none of which had reported results. We found that one trial (PEACE 1, NCT0195743), employing a factorial design, and also examining the effects of prostate radiotherapy, was not due to complete and publish for some years. The other 2 trials had completed recruitment, were due to report in 2017 [17,18], and each was large and individually adequately powered. Together, therefore, they would provide a large absolute information size. Based on accrual figures available at the time, we estimated they would represent 90% of all men randomised to abiraterone, and 70% of men randomised to abiraterone with or without docetaxel, also providing a large relative information size. Hence, rather than waiting for PEACE 1 results, we planned a potentially definitive meta-analysis of the 2 trials to coincide with the emergence of their results (PROSPERO protocol CRD42017058300). With information from trial protocols and investigators, we judged both trials at low risk of bias [19] for randomisation sequence generation, allocation concealment, blinding, completeness of outcome data, and selective provision of outcomes [20]. Collaborating trialists provided aggregate data in advance of publishing their own results [17,18], allowing us to complete and publish the systematic review in a similar time frame [20].

**Fig 3:**
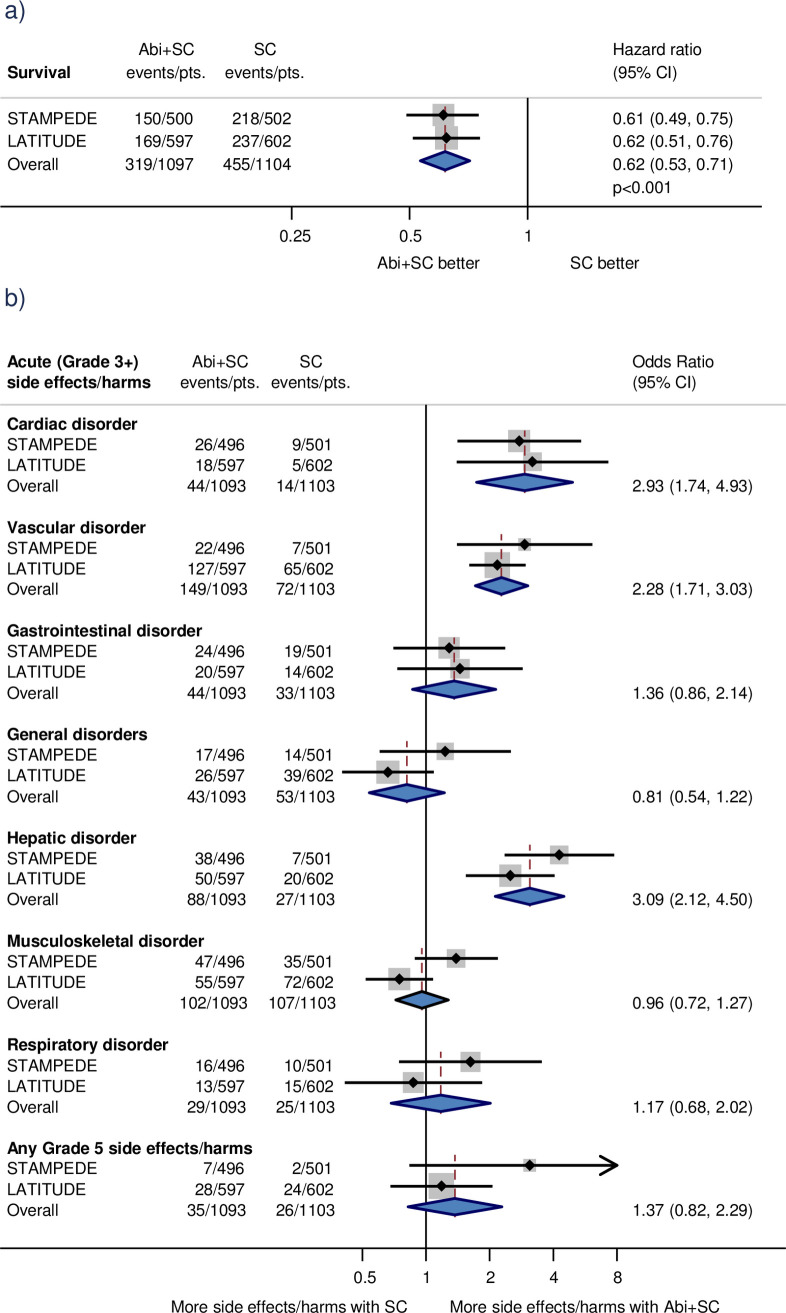
Effects of adding abiraterone to standard care in metastatic prostate cancer. Effect of adding abiraterone to standard care on (a) overall survival and (b) side effects/harms for men with metastatic prostate cancer. Each filled square denotes the HR or Peto OR for the trial comparison, with the horizontal lines showing the 95% CI. The size of the square is directly proportional to the amount of information contributed by a trial. The diamond represents a (fixed-effect) meta-analysis of the trial HRs/ORs, with the centre of this diamond indicating the HR/OR, and the extremities the 95% CI. Abi, Abiraterone; CI, confidence interval; HR, hazard ratio; OR, odds ratio; pts, participants; SC, Standard care.

The meta-analysis showed a substantial and convincing improvement in overall survival with the addition of abiraterone (Fig 3), equivalent to an absolute improvement of 14% at 3 years. Although the results of the included trials were conclusive in their own right, we were able to confine the meta-analysis to men with metastatic disease, making the results easier to interpret, and demonstrate remarkable consistency of effects across the trials. Also, we obtained extra results that allowed us to show that the effects of abiraterone did not vary across most predefined subgroups, and that although abiraterone increases some serious harms, it does not appear to be associated with excess mortality (Fig 3) [20].

Ultimately, fewer men than planned were recruited to PEACE 1, meaning the meta-analysis results were based on 82% of eligible men rather than the 70% anticipated [20]. This higher relative information size, paired with a robust meta-analysis, gave us confidence that the inclusion of PEACE 1 could not alter the direction or magnitude of the meta-analysis effect (although precision or heterogeneity might change by a small degree). Thus, instead of updating the aggregate data meta-analysis, we are collecting IPD to explore more thoroughly potential effect modifiers, and to compare reliably the effects of abiraterone with other treatments using network meta-analysis.

### 2. Effects of adding prostate radiotherapy to standard care in metastatic prostate cancer

In early 2016, we identified 3 trials investigating the addition of prostate radiotherapy to standard care for metastatic prostate cancer. One was still recruiting and not due to report for some time (PEACE 1, NCT01957436). The other two were due to report in late 2018, and we estimated that together they would comprise approximately 90% of eligible men and would provide 66% or 99% power to detect 5% (hazard ratio (HR) = 0.85) or 10% (HR = 0.72) absolute differences in 3-year survival, respectively. Thus, the anticipated absolute and relative information from the 2 trials was deemed sufficient for a definitive meta-analysis (PROSPERO protocol CRD42018096108). Both trials were judged to have low risk of bias for randomisation sequence generation, allocation concealment, completeness of outcome data, and provision of outcome data [21].

**Fig 4:**
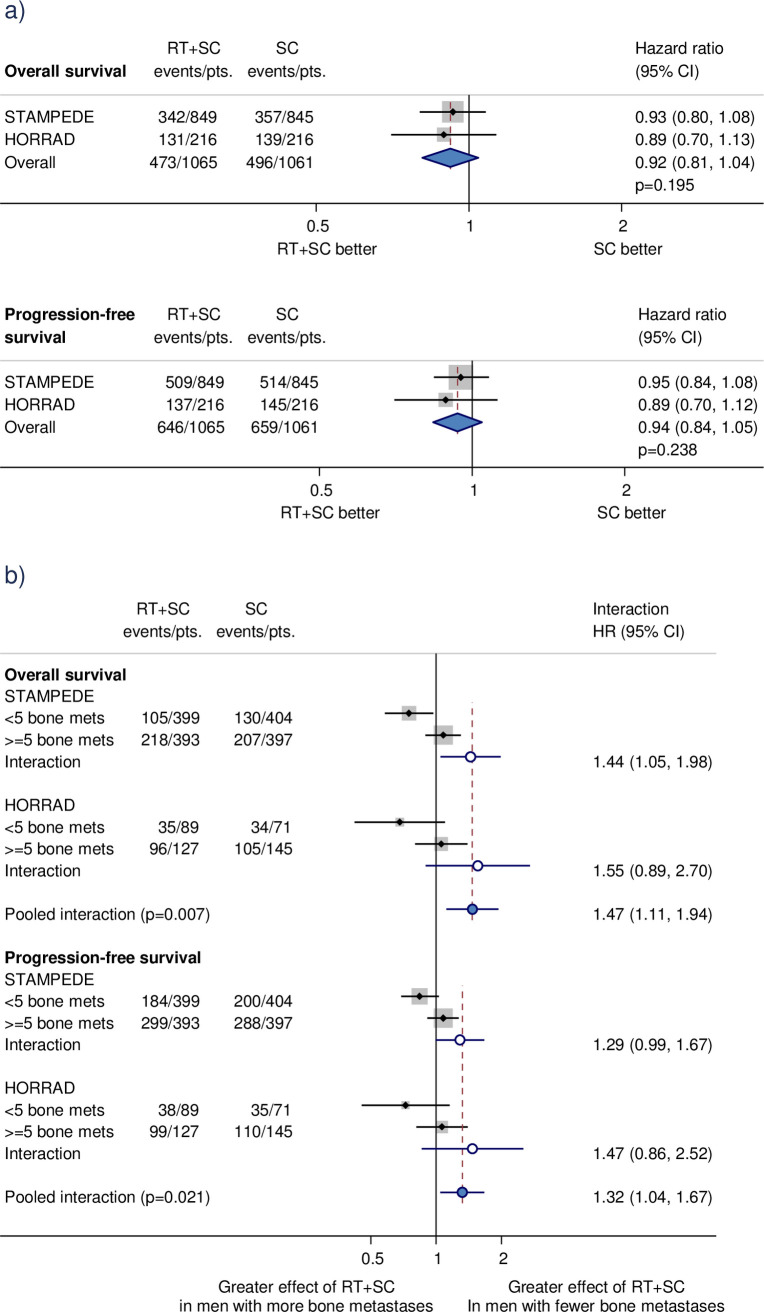
Effects of adding prostate radiotherapy to standard care in metastatic prostate cancer. Effect of adding prostate radiotherapy to standard care on survival and progression-free survival in all men (a) and by the number of bone metastases (b). For (a), labels and conventions are as per Fig 3. For (b), each filled square denotes the HR for each subgroup of men defined by the number of bone metastases. The size of the square is directly proportional to the amount of information contributed by a subgroup. Each filled circle denotes the HR for the within-trial interaction [36] between the effect of radiotherapy and the number of bone metastases, with the horizontal lines showing the 95% CI. The size of each circle is directly proportional to the amount of information contributed by a trial. The open circle represents a (fixed-effect) meta-analysis of the interaction HRs, with the horizontal line showing the 95% CI. CI, confidence interval; HR, hazard ratio; pts, participants; RT, prostate radiotherapy; SC, Standard care.

Adding prostate radiotherapy to standard care led to substantial improvements in biochemical progression and failure-free survival (Fig 4) [21]. While there was no clear evidence that prostate radiotherapy improved survival or progression-free survival in the overall population, these effects were influenced by the number of bone metastases (Fig 4) [21]. For men with few bone metastases, we found a 7% absolute improvement in survival at 3 years. Prospectively planning the subgroup analyses, obtaining the results necessary to conduct these, and demonstrating an interaction that was consistent across trials and outcomes and therefore unlikely to have arisen by chance, was a major strength.

### 3. Effects of immediate adjuvant versus early salvage prostate radiotherapy in localised prostate cancer

In 2014, we identified 3 ongoing trials of immediate adjuvant versus early salvage prostate radiotherapy for localised prostate cancer. As none were individually powered for survival, we initiated a PMA of IPD that might allow us to detect an effect on this outcome. However, recognising that it would be many years before data would mature and IPD would become available, we began planning a series of prospective aggregate data meta-analyses, each synthesising the results of an intermediate outcome when the required absolute information size was reached, starting with event-free survival.

We agreed a standardised definition of event-free survival with the trial investigators that would be applicable across the somewhat different trial designs. We anticipated that by autumn 2019, approximately 240 events would have occurred across the 3 trials, giving at least 90% power to detect a 5% difference in 5-year event-free survival (HR = 0.57). This, and the ability to obtain results based on 100% of eligible men, prompted us to plan a meta-analysis of this outcome (PROSPERO protocol CRD42019132669). The 3 trials were assessed to be at low risk of bias [22] for the randomisation process, and by obtaining extra results, we were able to limit biases associated with deviations from intended interventions, missing outcome data, measurement of the outcome and selection of the reported result [23].

Based on more events than predicted (270), the meta-analysis showed that immediate radiotherapy does not provide superior event-free survival (1% absolute difference at 5 years) [23]. As it is highly unlikely that such a small difference would translate to a later survival benefit, we could recommend an early salvage treatment policy and spare many men the side effects associated with immediate radiotherapy. The results of the meta-analysis were published [23] contemporaneously with the results of the 3 trials [24–26].

## Discussion

We have demonstrated that planning aggregate data meta-analysis prospectively and collaboratively using FAME produced timely evaluations of treatment effects that were less prone to bias than standard approaches. Working with trial investigators gave access to better quality aggregate data, allowing more consistent, reliable, and thorough analyses than are usually possible. It also enabled the meta-analysis results to be published in the same time frame as included trials, potentially increasing the visibility and impact of each.

FAME is suited to situations where quick and robust answers are needed, but prospective IPD meta-analysis would be too protracted. Nevertheless, the collaborative nature of FAME brings advantages more often associated with the IPD approach [6–8], such as inclusion of unreported results, harmonisation of outcomes, analysis of participant subgroup effects and wider endorsement and dissemination of results, as well as better identification of trials. If trial searches can be extended to a broad topic area, they can provide an overview of all interventions that have and will be evaluated. This allows strategic and prospective planning of multiple FAME reviews, which can be reprioritised as the status of trials change, and their analysis and dissemination plans evolve. If ongoing trials are identified after the initiation of a FAME review, they can be factored into meta-analysis planning, provided results of all or most eligible trials remain unknown, and any found later can be accounted for in the interpretation of meta-analysis results. If a definitive meta-analysis result is obtained, it can be used by trial investigators and independent data monitoring committees to inform decisions about continuing or adapting such ongoing trials.

As FAME makes use of aggregate data, it is best suited to synthesising overall effects of interventions and variations in effects across a number of predefined subgroups. IPD may be required for a more thorough investigation of potential effect modifiers, other detailed or complex analyses [8], or to ensure reliable estimates of effect [16]. However, a FAME review can help to justify the IPD approach, indicate which trials are most critical to include, and establish collaborations with investigators that will expedite subsequent data collection.

Predicting information size and determining the precise timing of reliable meta-analysis may be more challenging if eligible trials are numerous, cover a broad time span, are of short duration, or information on them is limited. This further emphasises the need to engage with trialists at an early stage, and to work closely with them to plan and conduct the meta-analysis, taking trial developments into account. Systematic reviewers may be concerned about the feasibility of liaising with investigators and the resource implications of FAME compared to a standard review. Certainly, it necessitates greater preplanning, as well as careful management, in order to avoid jeopardising individual trials, respect their publication timelines, and recognise the contribution of trial teams through coauthorship. Also, Trial Steering Committees may need to sanction participation, and nondisclosure or data sharing agreements may be required to protect information and results. However, we believe that all of this is achievable with a broadly similar level of funding and personnel. While involvement of trial investigators can hamper the objectivity of retrospective systematic reviews, it should not affect a prospective FAME approach, particularly if the systematic review team leads the design and conduct.

Pinpointing the timing of reliable meta-analysis based on information size and taking account of all trials whether they are included or not are key features of FAME. Although it has already been proposed that (absolute) information size should be optimised to ensure robust meta-analysis conclusions [27], and that it could be used to monitor accumulating evidence and account for multiple testing in cumulative pairwise [27,28] and network meta-analyses [29], these approaches seem to have been applied only to existing, retrospectively-planned systematic reviews (e.g., [28–30]).

To our knowledge, FAME represents the first prospective and collaborative approach to aggregate data meta-analysis. Similar PMAs are being employed to balance speed with rigour in the evaluation of COVID-19 therapies [31] (e.g., corticosteroids [32]). Using FAME to anticipate when enough information has accrued could add value in such settings, where many trials are being conducted quickly, and timeliness is vital. Instead, “living” systematic reviews [11] and “living” network meta-analyses [33] aim to incorporate new trial evidence as it emerges, but this means there is a risk that the information size of particular treatment comparisons is limited, and the results potentially unreliable. That said, living network meta-analysis has produced a snapshot of the available evidence on COVID 19 treatments [34], closely aligned to “living” guideline development [35]. Therefore, it could be advantageous to link with clinical guideline developers during the planning and conduct of FAME reviews.

## Conclusions

FAME can reduce the potential for bias, and produce more timely, thorough and reliable systematic reviews of aggregate data.

### Patient and public involvement

Patients or the public were not involved in the design, or conduct, or reporting, or dissemination plans of our research.

## Abbreviations

FAME: framework for adaptive meta-analysis
IPD: individual participant data
MA: prospective meta-analysis

## References

[1] Dickersin K. Publication bias: recognising the problem, understanding its origins and scope, and preventing harm. In: Rothstein H, Sutton A, Borenstein M, editors. Publication Bias in Meta-Analysis: Prevention, Assessment and Adjustments. Chichester: John Wiley & Sons Ltd; 2005. p. 261–86.

[2] Page MJ, Shamseer L, Altman DG, Tetzlaff J, Sampson M, Tricco AC, et al. Epidemiology and reporting characteristics of systematic reviews of biomedical research: A cross-sectional study. PLoS Med. 2016;13(5):e1002028. 10.1371/journal.pmed.1002028 27218655 PMC4878797

[3] Baudard M, Yavchitz A, Ravaud P, Perrodeau E, Boutron I. Impact of searching clinical trial registries in systematic reviews of pharmaceutical treatments: methodological systematic review and reanalysis of meta-analyses. BMJ. 2017;356:j448. 10.1136/bmj.j448 28213479 PMC5421496

[4] Garner P, Hopewell S, Chandler J, MacLehose H, Schunemann EAA, Beyene J, et al. When and how to update systematic reviews: consensus and checklist. BMJ. 2016;354. 10.1136/bmj.i3507 PMC4955793 27443385

[5] Ioannidis J. Next-generation systematic reviews: prospective meta-analysis, individual-level data. networks and umbrella reviews Br J Sports Med. 2017;51(20):1456–8. 10.1136/bjsports-2017-097621 28223307

[6] Seidler AL, Hunter KE, Cheyne S, Ghersi D, Berlin JA, Askie L. A guide to prospective meta-analysis. BMJ. 2019;367:l5342. 10.1136/bmj.l5342 31597627

[7] Stewart LA, Clarke MJ. on behalf of the Cochrane Working Party Group on Meta-analysis using Individual Patient Data. Practical methodology of meta-analyses (overviews) using updated individual patient data. Stat Med. 1995;14:2057–79. 10.1002/sim.4780141902 8552887

[8] Tierney JF, Vale CL, Riley R, Tudur Smith C, Stewart LA, Clarke M, et al. Individual participant data (IPD) meta-analyses of randomised controlled trials: Guidance on their use. PLoS Med. 2015;12(7):e1001855. 10.1371/journal.pmed.1001855 26196287 PMC4510878

[9] Tierney JF, Vale CL, Burdett S, Fisher D, Rydzewska LHM, Parmar MKB. Timely and reliable evaluation of the effects of interventions: a framework for adaptive meta-analysis (FAME). Trials. 2017;18(Suppl. 1):P351.

[10] Thomas J, Askie LM, Berlin JA, Elliott J, Ghersi D, Simmonds M, et al. Prospective approaches to accumulating evidence. In: Higgins JPT, Thomas J, Chandler J, Cumpston M, Li T, Page MJ, et al., editors. Cochrane Handbook for Systematic Reviews of Interventions. London: Cochrane; 2019.

[11] Elliott JH, Synnot A, Turner T, Simmonds M, Akl EA, McDonald S, et al. Living systematic review: 1. Introduction-the why, what, when, and how. J Clin Epidemiol. 2017;91:23–30. 10.1016/j.jclinepi.2017.08.010 28912002

[12] James ND, Sydes MR, Clarke NW, Mason MD, Dearnaley DP, Spears MR, et al. Addition of docetaxel, zoledronic acid, or both to first-line long-term hormone therapy in prostate cancer (STAMPEDE): survival results from an adaptive, multiarm, multistage, platform randomised controlled trial. Lancet. 2016;387(10024):1163–77. 10.1016/S0140-6736(15)01037-5 26719232 PMC4800035

[13] Vale CL, Vale CL, Burdett S, Rydzewska LH, Albiges L, Clarke NW. Addition of docetaxel or bisphosphonates to standard of care in men with localised or metastatic, hormone-sensitive prostate cancer: a systematic review and meta-analyses of aggregate data. Lancet Oncol. 2016;17(2):243–56. 10.1016/S1470-2045(15)00489-1 26718929 PMC4737894

[14] Lefebvre C, Glanville J, Briscoe S, Littlewood A, Marshall C, Metzendorf M-I, et al. Chapter 4: Searching for and selecting studies. In: Higgins JPT, Thomas J, Chandler J, Cumpston M, Li T, Page MJ, et al., editors. Cochrane Handbook for Systematic Reviews of Interventions version 60 (updated July 2019) Cochrane, 2019 Available from: wwwtrainingcochraneorg/handbook.

[15] Vale CL, Tierney JF, Burdett S. Can trial quality be reliably assessed from published reports of cancer trials: evaluation of risk of bias assessments in systematic reviews. BMJ. 2013;346:f1798. 10.1136/bmj.f1798 23610376

[16] Tierney JF, Fisher DJ, Burdett S, Stewart LA, Parmar MKB. Comparison of aggregate and individual participant data approaches to meta-analysis of randomised trials: An observational study. PLoS Med. 2020;17(1):e1003019. 10.1371/journal.pmed.1003019 32004320 PMC6993967

[17] James ND, de Bono JS, Spears MR, Clarke NW, Mason MD, Dearnaley DP, et al. Abiraterone for prostate cancer not previously treated with hormone therapy. N Engl J Med. 2017;377(4):338–51. 10.1056/NEJMoa1702900 28578639 PMC5533216

[18] Fizazi K, Tran N, Fein L, Matsubara N, Rodriquez-Antolin A, Alekseev BY, et al. Abiraterone plus prednisone in metastatic castration-sensitive prostate cancer. N Engl J Med. 2017(4):352–60. 10.1056/NEJMoa1704174 28578607

[19] Higgins JP, Altman DG, Gotzsche PC, Juni P, Moher D, Oxman AD, et al. The Cochrane Collaboration’s tool for assessing risk of bias in randomised trials. BMJ. 2011;d5928:343. 10.1136/bmj.d5928 22008217 PMC3196245

[20] Rydzewska LHM, Burdett S, Vale CL, Clarke NW, Fizazi K, Kheoh T, et al. Adding abiraterone to androgen deprivation therapy in men with metastatic hormone-sensitive prostate cancer: A systematic review and meta-analysis. Eur J Cancer. 2017;84:88–101. 10.1016/j.ejca.2017.07.003 28800492 PMC5630199

[21] Burdett S, Boeve LM, Ingleby FC, Fisher DJ, Rydzewska LH, Vale CL, et al. Prostate radiotherapy for metastatic hormone-sensitive prostate cancer: a STOPCAP systematic review and meta-analysis. Eur Urol. 2019;76(1):115–24. 10.1016/j.eururo.2019.02.003 30826218 PMC6575150

[22] Sterne JAC, Savovic J, Page MJ, Elbers RG, Blencowe NS, Boutron I, et al. RoB 2: a revised tool for assessing risk of bias in randomised trials. BMJ. 2019;366:I4989. 10.1136/bmj.l4898 31462531

[23] Vale CL, Fisher D, Kneebone A, Parker C, Pearse M, Richaud P, et al. Adjuvant or early salvage radiotherapy for the treatment of localised and locally advanced prostate cancer: a prospectively planned systematic review and meta-analysis of aggregate data. Lancet. 2020;396(10260):1422–31. 10.1016/S0140-6736(20)31952-8 33002431 PMC7611137

[24] Parker CC, Clarke NW, Cook AD, Kynaston HG, Petersen PM, Catton C, et al. Timing of radiotherapy after radical prostatectomy (RADICALS-RT): a randomised, controlled phase 3 trial. Lancet. 2020;396(10260):1413–21. 10.1016/S0140-6736(20)31553-1 33002429 PMC7616947

[25] Kneebone A, Fraser-Browne C, Duchesne GM, Fisher R, Frydenberg M, Herschtal A, et al. Adjuvant radiotherapy versus early salvage radiotherapy following radical prostatectomy (TROG 08.03/ANZUP RAVES): a randomised, controlled, phase 3, non-inferiority trial. Lancet Oncol. 2020;21(10):1331–40. 10.1016/S1470-2045(20)30456-3 33002437

[26] Sargos P, Chabaud S, Latorzeff I, Magne N, Benyoucef A, Supiot S, et al. Adjuvant radiotherapy versus early salvage radiotherapy plus short-term androgen deprivation therapy in men with localised prostate cancer after radical prostatectomy (GETUG-AFU 17): a randomised. phase 3 trial. Lancet Oncol. 2020;21(10):1341–52. 10.1016/S1470-2045(20)30454-X 33002438

[27] Pogue JM, Yusuf S. Cumulating evidence from randomized trials: utilizing sequential monitoring boundaries for cumulative meta-analysis. Control Clin Trials. 1997;18(6):580–93. discussion 661–6. 10.1016/s0197-2456(97)00051-2 9408720

[28] Wetterslev J, Thorlund K, Brok J, Gluud C. Trial sequential analysis may establish when firm evidence is reached in cumulative meta-analysis. J Clin Epidemiol. 2008;61(1):64–75. 10.1016/j.jclinepi.2007.03.013 18083463

[29] Nikolakopoulou A, Mavridis D, Egger M, Salanti G. Continuously updated network meta-analysis and statistical monitoring for timely decision-making. Stat Methods Med Res. 2018;27(5):1312–30. 10.1177/0962280216659896 27587588 PMC5863798

[30] Imberger G, Thorlund K, Gluud C, Wetterslev J. False-positive findings in Cochrane meta-analyses with and without application of trial sequential analysis: an empirical review. BMJ Open. 2016;6(8):e011890. 10.1136/bmjopen-2016-011890 27519923 PMC4985805

[31] Naci H, Kesselheim AS, Rottingen JA, Salanti G, Vandvik PO, Cipriani A. Producing and using timely comparative evidence on drugs: lessons from clinical trials for covid-19. BMJ. 2020;371:m3869. 10.1136/bmj.m3869 33067179

[32] WHO Rapid Evidence Appraisal for COVID-19 Therapies Working Group, Sterne JAC, Murthy S, Diaz JV, Slutsky AS, Villar J, et al. Association Between Administration of Systemic Corticosteroids and Mortality Among Critically Ill Patients With COVID-19: A Meta-analysis. JAMA. 2020;324(13):1330–41. 10.1001/jama.2020.17023 32876694 PMC7489434

[33] Crequit P, Trinquart L, Yavchitz A, Ravaud P. Wasted research when systematic reviews fail to provide a complete and up-to-date evidence synthesis: the example of lung cancer. BMC Med. 2016;14:8. 10.1186/s12916-016-0555-0 26792360 PMC4719540

[34] Siemieniuk RA, Bartoszko JJ, Ge L, Zeraatkar D, Izcovich A, Kum E, et al. Drug treatments for covid-19: living systematic review and network meta-analysis. BMJ. 2020;m2980:370. 10.1136/bmj.m2980 32732190 PMC7390912

[35] Lamontagne F, Agoritsas T, Macdonald H, Leo YS, Diaz J, Agarwal A, et al. A living WHO guideline on drugs for covid-19. BMJ. 2020;m3379:370. 10.1136/bmj.m3379 32887691

[36] Fisher DJ, Carpenter JR, Morris TP, Freeman SC, Tierney JF. Meta-analytical methods to identify who benefits most from treatments: daft, deluded, or deft approach? BMJ. 2017;356:j573. 10.1136/bmj.j573 28258124 PMC5421441

